# Beyond Nightmares: How Sleep Issues Might Trigger Post-Traumatic Stress Disorder

**DOI:** 10.1192/j.eurpsy.2025.1954

**Published:** 2025-08-26

**Authors:** O. Inanc

**Affiliations:** Gulhane Training and Research Hospital, Ankara, Türkiye

## Abstract

**Introduction:**

Post-traumatic stress disorder (PTSD) is a psychiatric condition that arises after exposure to traumatic events and is characterized by intrusive memories, emotional dysregulation, and hyperarousal. Sleep disturbances, such as insomnia and disrupted circadian rhythms, are often reported in PTSD. However, recent studies suggest that sleep disorders may not only be a consequence of PTSD but could also contribute to its development (DeViva, 2021; Seo et al., 2022). Sleep is crucial in emotional regulation, memory consolidation, and stress recovery. Disruptions in sleep patterns may impair these processes, increasing vulnerability to psychiatric conditions such as PTSD (Zhou, 2023). This systematic review evaluates current evidence on whether sleep disturbances serve as a precursor or risk factor for PTSD across various populations. Understanding the role of sleep disturbances in the onset of PTSD is critical for developing early intervention and prevention strategies.

**Objectives:**

This systematic review aims to evaluate whether sleep disturbances act as a risk factor for the development of PTSD by synthesizing recent evidence from diverse populations.

**Methods:**

A comprehensive literature search was conducted using the keywords “PTSD” and “sleep disturbance” in the database on PubMed. Out of 143 studies published within the last five years, eight articles that specifically examined sleep disturbances as a risk factor for PTSD were selected for this review.

**Results:**

The review of selected studies highlights a strong association between sleep disorders and an increased risk of PTSD development across various populations. Poor sleep quality was associated with a 60% greater likelihood of developing PTSD. In a nationally representative sample of U.S. veterans, over 22% reported poor sleep quality, which was a significant predictor of PTSD. (DeViva, 2021). In a study of 67,905 college students, those with sleep disturbances had significantly higher rates of PTSD (52.3% vs. 33.0%) and depression (47.7% vs. 13.2%). (Wang et al., 2022). Moreover, emotional dysregulation, often exacerbated by sleep disorders, was found to aggravate PTSD symptoms further. The studies also indicated that interventions targeting sleep disorders, such as cognitive behavioral therapy for insomnia, not only improved sleep quality but also contributed to a reduction in trauma-related symptoms, suggesting potential for PTSD prevention (Ranney, 2022).

**Conclusions:**

This review highlights that sleep disturbances are a significant risk factor for the development of PTSD. Early identification and treatment of sleep disorders could serve as a preventative strategy for individuals exposed to trauma. Future research should explore the underlying mechanisms of this relationship and the effectiveness of sleep-focused interventions in reducing PTSD risk.

**Disclosure of Interest:**

None Declared

